# Pairwise Paternity Assignment With Forward–Backward Simulations: Refining CERVUS Using Trio‐Based Likelihood and Locus‐Specific Error Rates

**DOI:** 10.1002/ece3.72230

**Published:** 2025-09-27

**Authors:** Mahmoud Amiri Roudbar, Seyedeh Fatemeh Mousavi, Mahdi Akbarzadeh, Sabrina H. Brounts, Mehdi Momen

**Affiliations:** ^1^ Department of Animal Science Safiabad‐Dezful Agricultural and Natural Resources Research and Education Center, Agricultural Research, Education and Extension Organization (AREEO) Dezful Iran; ^2^ Department of Animal Breeding and Genetics Swedish University of Agricultural Sciences Uppsala Sweden; ^3^ Cellular and Molecular Endocrine Research Center, Research Institute for Endocrine Sciences, Shahid Beheshti University of Medical Sciences Tehran Iran; ^4^ Department of Surgical Sciences, School of Veterinary Medicine University of Wisconsin‐Madison Madison Wisconsin USA

**Keywords:** genealogical relationships, Pairwise assignment algorithm, paternity analysis, STR marker

## Abstract

Highly polymorphic markers like microsatellites are extensively utilized in genomic studies to analyze and infer genealogical relationships among individuals in a population. Traditional methods to identify the most likely parent among the potential known candidates rely on a single hypothetical distribution derived from population parameters. Indeed, these methods often make simplifying assumptions, such as a homogeneous genetic structure, consistent typing error rates across all genomic loci, and even random allele substitutions based on allele frequencies, which are frequently violated in practical applications. In this study, we introduce an enhanced likelihood‐based approach, called the “Pairwise” algorithm, which builds on the widely used CERVUS method by calculating a trio‐specific significance criterion for each father–mother–offspring combination using forward and backward simulations. Our method also accounts for the variable typing errors across genomic loci to enhance the accuracy of paternity analysis. Our findings showed that employing the Pairwise algorithm increases the power of paternity assignments by reducing the number of falsely assigned parents. Furthermore, adjusting likelihood equations to accommodate variable typing errors significantly improves the accuracy of paternity assignments. The developed approach represents a significant advancement in paternity analysis by addressing the limitations of traditional approaches. These improvements have the potential to significantly impact genealogical research and related fields, providing a more robust framework for analyzing complex genetic relationships in the context of parent assignment. Future research should focus on further refining this method and exploring its applications in diverse populations and genetic contexts.

## Graphical abstract

This study introduces a novel “Pairwise” assignment method for paternity analysis that improves accuracy by simulating likelihoods for each parent–offspring trio while accounting for variable typing errors across genomic loci. The approach outperforms traditional methods by reducing false assignments and better handling genetic complexity, offering a more robust framework for genealogical inference.

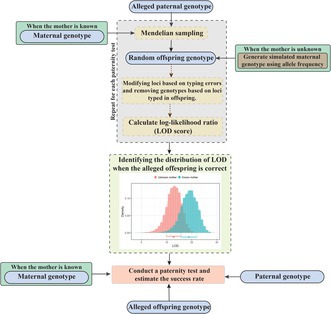

## 1 Introduction

Genealogical relationships among individuals have provided remarkable insights into the reproductive lives and population structures of plants and animals (Castro et al. 2004; Leroy 2011; Sneller 1994). Additionally, pedigree‐derived information is crucial for the accurate estimation of quantitative genetic parameters in genetic evaluations. The accuracy of relationship information is a critical factor, as it can significantly impact the estimation of population parameters such as inbreeding coefficients, genetic variability, and effective population size (Lehocká et al. 2021; Nishio et al. 2023), and also individual assessments like the estimated breeding value (Yang and Su 2016). By analyzing DNA information, it is possible to identify and correct errors in pedigree, such as misattributed parentage, and improve the pedigree's quality. The DNA‐based approaches provide a robust framework for verifying historical records, ensuring that pedigrees are more precise and reliable. Additionally, genetic data can uncover previously unknown relationships and provide more complete genealogical information. Consequently, integrating genetic information into genealogical studies not only rectifies inaccuracies but also improves prediction quality (Velazco et al. 2019).

Among all the discovered genetic markers, short tandem repeat (STR, also known as microsatellite) revolutionized parentage analysis by providing highly polymorphic and codominant genetic variation necessary for accurately distinguishing between individuals (Pemberton 2008). So far, using STR information, four types of parentage analysis techniques were initially described by Jones and Ardren (2003): simple exclusion, categorical allocation, fractional allocation, and parental reconstruction. This framework was later expanded to include six categories by Jones et al. (2010), with the addition of full probability parentage analysis and sibship reconstruction. Current methodologies for parentage analysis are relatively simplistic, often predicated on several assumptions which are involved: a population with a homogeneous genetic structure, consistent typing error rates across all loci, and random allele substitutions based on population frequencies due to mutation (Christie 2010; Gerber et al. 2003; Kalinowski et al. 2007; Marshall et al. 1998). An exception is COLONY, which can accommodate and re‐estimate locus‐specific error rates during analysis (Jones and Wang 2010). However, these assumptions are frequently violated in practical applications due to the variability in genotyping error rates across different loci, the occurrence of allelic preference substitutions (e.g., systematic stutter or dropout bias), rather than purely random substitutions, and nonrandom mating patterns (Bonin et al. 2004; Creel et al. 2003; Huang et al. 2018; Slate et al. 2000). Additionally, it has been shown that incorporating inbreeding coefficients into parentage estimation models can mitigate the impact of population structure (Huang et al. 2018). Programs such as COLONY provide robust full‐pedigree/sibship reconstruction and models locus‐specific error rates, which are powerful when comprehensive sampling and pedigree inference are desired (Jones and Wang 2010). However, COLONY is computationally intensive and its design is optimized for pedigree‐wide inference, making it less practical for studies focused on testing a limited number of candidate parents or cases where maternal genotypes are missing. There remains a gap for lightweight, trio‐based methods that (a) remain within the classical likelihood‐odds ratio (LOD) paradigm, (b) compute trio‐specific significance thresholds for each father–mother–offspring combination, and (c) explicitly incorporate locus‐specific (variable) typing errors directly into the LOD calculation. The Pairwise approach introduced here is designed to fill this gap, delivering case‐specific thresholds via forward/backward simulations and improving control of false assignments, particularly when the genotype of one parent is missing (i.e., when only the presumed parent and offspring genotypes are available).

The approach proposed by Marshall et al. (1998) has gained widespread popularity in recent years. This approach was subsequently refined by Kalinowski et al. (2007) to more accurately calculate the probability of observing erroneous genotypes. Their method involves selecting the most likely parent from a group of nonexcluded putative parents based on the likelihood of paternity, accounting for genotyping errors. The identification of a significant threshold is a critical step in this method. To achieve this, an innovative simulation technique was employed to estimate the Δ distribution representing the disparity in LOD scores between the most probable father, is indicated by the highest LOD score, and the second most likely candidate. The Δ distribution is calculated for the population, and all paternity assignments are evaluated for significance using a single Δ distribution. It is more practical to consider that each parentage test for a father–mother–offspring trio has its own hypothesis distribution. This is due to the alleles carried by the alleged and/or known parent and inherited by the offspring differing in each test case, with allele frequency being a crucial component of LOD score calculation, consequently affecting the real Δ distribution for each case. In other words, the Δ distribution can vary based on the alleles present in each trio, especially if a specific simulation is conducted based on these genotypes. This situation becomes more complex when there are missing genotypes in the parents and/or the offspring. Therefore, we demonstrate that case‐by‐case simulation for calculating critical values can improve the accuracy of paternity testing or assignment in certain aspects. We employed forward and backward simulations to test our hypothesis for paternity testing and assignment, respectively. By utilizing variable genotyping errors across loci for LOD score calculation, we showed an improvement in the accuracy of estimating paternity.

## 2 Material and Methods

### 2.1 Likelihood Equations

The imperfection of genotyping data may lead to discrepancies at a single locus between the presumed parent and offspring. Such mismatches are anticipated as part of the genotyping process and do not necessarily result in the exclusion of paternity. In modeling genotyping errors, we adopted the framework initially proposed by Marshall et al. (1998). Consistent with their methodology, our model assumes that the occurrence of a genotyping error results in an observed genotype with a probability directly proportional to the genotype's frequency within the population and allows the error modeling to accurately reflect population‐based frequencies. This principle facilitates the practical application of the model to account for genotyping inaccuracies. While our model concurs with theirs in treating genotyping error rates as independent variables and constant across individuals, it differs by not presuming uniformity of these rates across different loci. In our model, we denote an observed genotype for *l*th locus (where $l=1,\ldots ,n_{l}$) by $g_{l}$, the genotyping error rate observed by $\varepsilon_{l}$, and the Hardy–Weinberg equilibrium frequency of genotype $g_{l}$ by $p\left(g_{l}\right)$. Within the framework of this genotyping error model, the probabilities of observing $g_{l}$ accurately and erroneously are $\left(1-\varepsilon_{l}\right)p\left(g_{l}\right)$ and $\varepsilon_{l}p\left(g_{l}\right)$, respectively. The sum of these two probabilities denotes the comprehensive likelihood of observing $g_{l}$.

The likelihood of *l*th genotype observed in a mother ($g_{l,m}$), alleged father ($g_{l,\mathrm{af}}$), and offspring ($g_{l,o}$) was calculated based on the framework initially introduced by Kalinowski et al. (2007). This model has been further refined and adapted to account for variability in error rates across different genomic loci. To assess whether the alleged father is indeed the correct father, we calculate the likelihood ratio. This ratio compares the probability of observing the genotypes under two contrasting hypotheses: H_1_: where the alleged father is the true father, and H_2_: where the alleged father is not the true father. Under the assumption that hypothesis H_1_ holds true, and given the known genotype of the mother, the likelihood (L) of observing $g_{l,m}$, $g_{l,a},$ and $g_{l,o}$ at *l*th locus is quantified as:

$$\begin{array}{c} L{\left(H_{1}\right)}_{l}=P\left(g_{l,m}\right)P\left(g_{l,\mathrm{af}}\right)\left[{\left(1-\varepsilon_{l}\right)}^{3}\right.T\left(g_{l,o}|g_{l,m},g_{l,\mathrm{af}}\right)+\varepsilon_{l}{\left(1-\varepsilon_{l}\right)}^{2}\left(T\left(g_{l,o}|g_{l,m}\right)+T\left(g_{l,o}|g_{l,\mathrm{af}}\right)+P\left(g_{l,o}\right)\right)+\varepsilon_{l}^{2}{\left(1-\varepsilon_{l}\right)}^{3}P\left(g_{l,o}\right)+\left.\varepsilon_{l}^{3},P,\left(g_{l,o}\right)\right] \end{array}$$

where *T*(·) represents standard Mendelian transition probabilities as defined previously in the model proposed by Marshall et al. (1998). Similarly, under identical presumptions, the likelihood equation for H_2_ is:

$$L{\left(H_{2}\right)}_{l}=P\left(g_{l,m}\right)P\left(g_{l,\mathrm{af}}\right)\left[{\left(1-\varepsilon_{l}\right)}^{3}T\left(g_{l,o}|g_{l,m}\right)+\varepsilon_{l}{\left(1-\varepsilon_{l}\right)}^{2}\left(T\left(g_{l,o}|g_{l,m}\right)+P\left(g_{l,o}\right)\right)+\varepsilon_{l}^{2}{\left(1-\varepsilon_{l}\right)}^{3}P\left(g_{l,o}\right)+\varepsilon_{l}^{3}P\left(g_{l,o}\right)\right]$$

In cases where the maternal genotype is unknown, the likelihood of H_1_ and H_2_ are modified accordingly:

$$L{\left(H_{1}\right)}_{l}=P\left(g_{l,\mathrm{af}}\right)\left[{\left(1-\varepsilon_{l}\right)}^{2}T\left(g_{l,o}|g_{l,\mathrm{af}}\right)+\varepsilon_{l}{\left(1-\varepsilon_{l}\right)}^{2}P\left(g_{l,o}\right)+\varepsilon_{l}^{2}P\left(g_{l,o}\right)\right]$$

and

$$L{\left(H_{2}\right)}_{l}=P\left(g_{l,\mathrm{af}}\right)\left[{\left(1-\varepsilon_{l}\right)}^{2}P\left(g_{l,o}\right)+\varepsilon_{l}{\left(1-\varepsilon_{l}\right)}^{2}P\left(g_{l,o}\right)+\varepsilon_{l}^{2}P\left(g_{l,o}\right)\right]$$

When considering joint paternity and maternity, the likelihoods for H_1_ and H_2_ are expressed as follows:

$$L{\left(H_{1}\right)}_{l}=P\left(g_{l,\mathrm{am}}\right)P\left(g_{l,\mathrm{af}}\right)\left[{\left(1-\varepsilon_{l}\right)}^{3}T\left(g_{l,o}|g_{l,\mathrm{am}},g_{l,\mathrm{af}}\right)+\varepsilon_{l}{\left(1-\varepsilon_{l}\right)}^{2}\left(T\left(g_{l,o}|g_{l,\mathrm{am}}\right)+T\left(g_{l,o}|g_{l,\mathrm{af}}\right)+P\left(g_{l,o}\right)\right)+\varepsilon_{l}^{2}{\left(1-\varepsilon_{l}\right)}^{3}P\left(g_{l,o}\right)+\varepsilon_{l}^{3}P\left(g_{l,o}\right)\right]$$

and

$$L{\left(H_{2}\right)}_{l}=P\left(g_{l,\mathrm{am}}\right)P\left(g_{l,\mathrm{af}}\right)\left[{\left(1-\varepsilon_{l}\right)}^{3}P\left(g_{l,o}\right)+\varepsilon_{l}{\left(1-\varepsilon_{l}\right)}^{2}P\left(g_{l,o}\right)+\varepsilon_{l}^{2}{\left(1-\varepsilon_{l}\right)}^{3}P\left(g_{l,o}\right)+\varepsilon_{l}^{3}P\left(g_{l,o}\right)\right]$$

where $g_{l,\mathrm{am}}$ represents the genotype observed in an alleged mother. The LOD, for each alleged father at *l*th locus were calculated as

$${\mathrm{LOD}}_{l}=\ln\left[\frac{L{\left(H_{1}\right)}_{l}}{L{\left(H_{2}\right)}_{l}}\right]$$

In a paternity analysis utilizing multiple independent genetic markers, the cumulative LOD score is computed by summing the LOD scores across all loci, $\mathrm{LOD}=\sum_{l=1}^{n_{l}}{\mathrm{LOD}}_{l}$. A positive LOD score suggests a higher probability of the tested male being the true father compared to a random male from the general population. Conversely, a negative cumulative LOD score implies a lower probability of paternity.

### 2.2 Determining Paternity Through LOD Scores

Marshall et al. (1998) introduced a discriminant statistic, denoted as Δ, for assessing paternity determination based on computed LOD scores across a cohort of potential fathers. If the model achieves a statistical significance level, it supports identifying the male with the highest LOD score as the true father. In this model, the allele frequencies ascertained within the studied population are employed to simulate the genotypes of the parents as well as additional unrelated potential males. To address population structure, the CERVUS algorithm provides two simulation options: (1) inbreeding (including selfing) of the true parents and (2) the simulation of relatives among the candidate parents. These options help to mitigate the bias in Δ estimates, which can lead to overestimation or underestimation for true and false parents, respectively. Without considering population structure, the critical Δ value may be underestimated, particularly in populations with high levels of relatedness or inbreeding. For instance, when a candidate male has a high additive genetic relationship with the offspring, the calculated Δ may be significantly smaller than in cases where the candidate male is unrelated. This bias increases the likelihood of incorrectly including false candidates in parentage assessments (Jones and Wang 2010; Marshall et al. 1998). Although the CERVUS algorithm incorporates allelic frequencies, inbreeding, and relationships among candidate parents, it most likely does not fully capture the genetic architecture of the population under study. Furthermore, in certain real‐world scenarios, information regarding inbreeding and relationships among candidate parents may not be available, exacerbating the situation. To address this issue, we introduce the Pairwise LOD simulation, which estimates paternity using exhaustive simulations based on parental genotypes were independent of the pedigree or relatedness structure among candidate parents. When one parent was unknown, the missing parental alleles were generated according to observed population allele frequencies, consistent with standard likelihood‐based methods (e.g., CERVUS). This method generates distributions of LOD scores for true parent–offspring pairs, enabling the determination of critical LOD thresholds that distinguish true parents from unrelated candidates with confidence. Two simulation types, backward and forward, are employed to analyze LOD distributions, facilitating paternity testing and assignment analysis. Details for implementing this methodology in parentage studies are provided in subsequent sections.

### 2.3 Forward Simulation for Paternity Test

**FIGURE 1:**
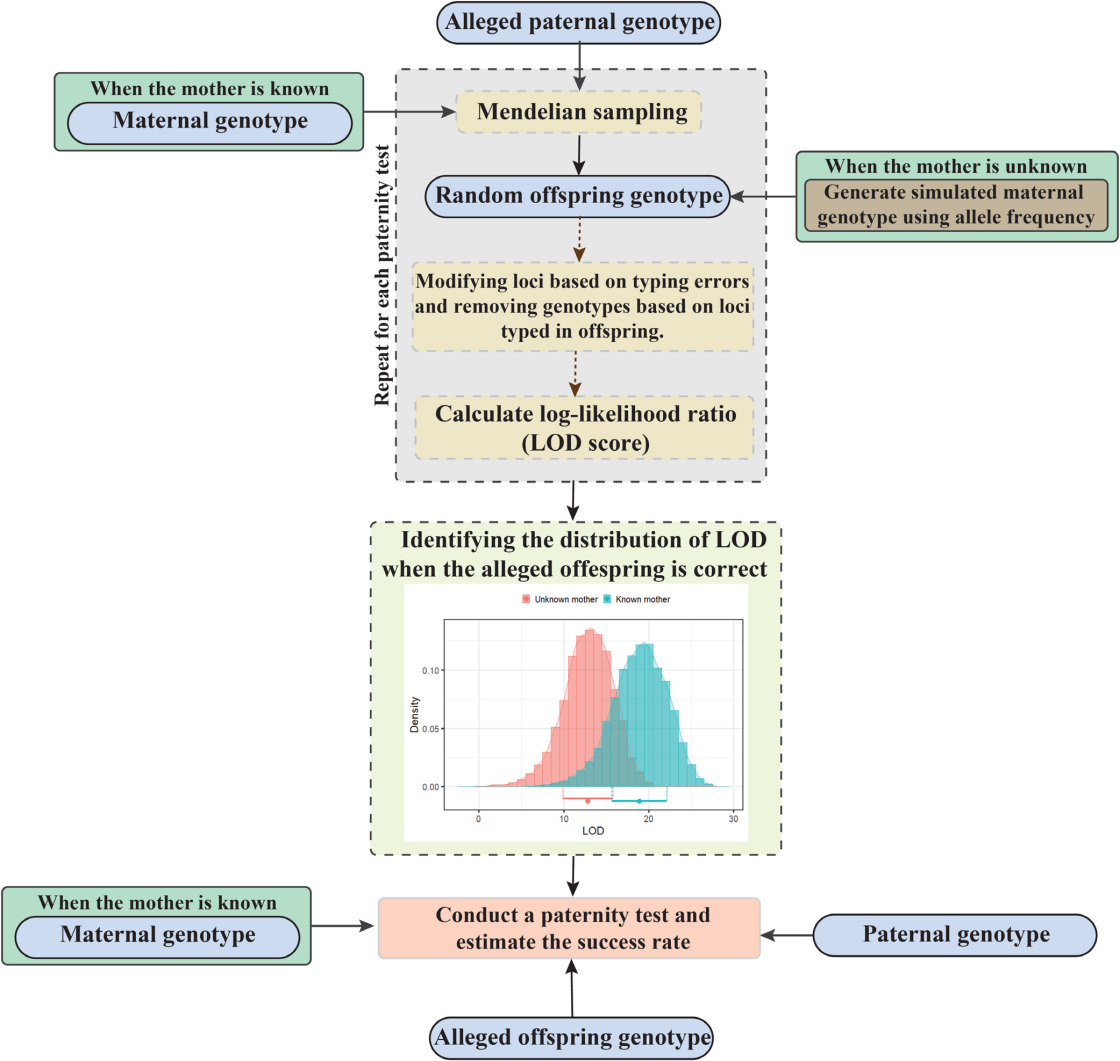
Flowchart illustrating the forward simulation procedure for paternity testing of an alleged father. Genotype information from the alleged father and mother (if available) is randomly sampled using Mendelian principles and modified based on specific parameters to generate a random offspring genotype. This sampling procedure is repeated to derive the distribution of the likelihood‐odds ratio (LOD) for the true father. This distribution is then utilized to conduct the paternity test.

In paternity analysis, the primary objective was to confirm whether the alleged father is the biological father. To achieve this, a forward simulation in multilocus population genetics was implemented to find the distribution of LOD scores for the true parents to use in the paternity test (Figure 1). An offspring genotype is derived by Mendelian sampling of the parental alleles. When only the alleged father's genotypes are available, the second alleles are generated from frequencies observed in the population. The simulated genotype for the true offspring is subsequently modified to account for incorrectly typed loci and missing loci. LOD scores are then computed for the alleged parent, which is indeed the true parent. This entire simulation process is iterated, and paternity tests are conducted on a substantial number of simulated offspring. After determining the LOD distribution for all possible true random offspring, a paternity test is conducted to verify if the alleged father is the true father, using a specific *p* value criterion.

### 2.4 Backward Simulation for Paternity Assignment

**FIGURE 2:**
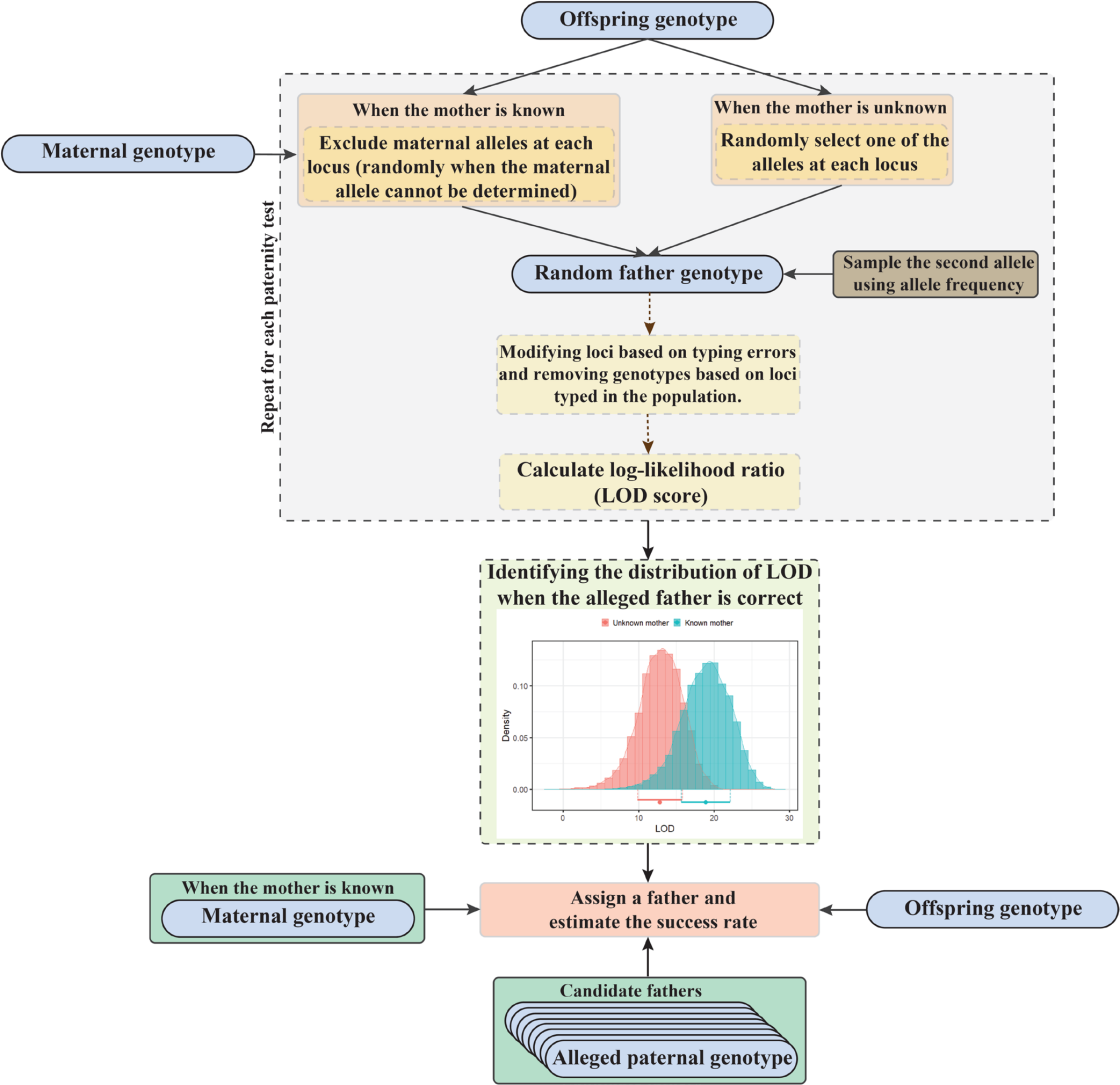
Flowchart illustrating the backward simulation procedure for assigning the true father among the candidate males. The first allele at each locus for the random true father is sampled from the offspring genotypes. When the mother is known, trackable maternal alleles are excluded from the offspring genotypes before sampling. The second allele is sampled based on the allele frequency of the population. The genotypes of the random father are then modified based on specific population parameters. This sampling procedure is repeated to derive the distribution of the likelihood‐odds ratio (LOD) for all possible random true fathers. This distribution is subsequently utilized to conduct the paternity assignment. In this study, we used 10,000 simulations to generate distributions of true LOD.

In contrast to paternity testing, assignment testing is conducted to identify the true father among candidate males. For this purpose, a backward simulation in multilocus population genetics is employed to determine the distribution of LOD scores for all possible true fathers, which is subsequently utilized in the assignment test (Figure 2). Initially, a random father genotype is derived through Mendelian sampling of the offspring alleles. When the genotype of the mother is known, the inherited allele from the mother is excluded if it can be tracked. In cases where the second alleles need to be generated, they are based on frequencies observed in the population. Subsequently, the simulated genotype for the true random father is adjusted to account for incorrectly typed loci and missing loci. LOD scores are then calculated for the random father. This entire simulation process is repeated, and assignment tests are performed on a large set of simulated fathers. After determining the LOD distribution for all possible true random fathers, a father assignment test is conducted to identify the true father among the candidate males using a specific *p* value criterion.

### 2.5 Genomic Data Simulation

To evaluate the performance of the proposed algorithm, we employed a comprehensive simulation approach to generate a population with short tandem repeats (STRs) or STR genotypes. We simulated 15 STR loci for everyone, each with 10 alleles. Each generation consisted of two sexes in equal proportions (0.5). The proportions of males and females contributing to the next generation were set at 0.5 and 0.8, respectively. We also considered overlapping generations, with a maximum of three generations for both parents. For these overlapping generations, we assigned proportions of 0.6, 0.3, and 0.1 to parents born one, two, and three generations ago, respectively. For instance, fathers in the simulated third generation included 0.3 (0.5 × 0.6), 0.15 (0.5 × 0.3), and 0.05 (0.5 × 0.1) proportions of males born in generations two, one, and zero, respectively. To model typing errors, we distinguished between mutation and genotyping error. The baseline mutation rate was set to 1 × 10^−5^, consistent with empirical estimates for STR mutation ranging from 1 × 10^−6^ to 1 × 10^−2^ (Bhargava and Fuentes 2010). However, mutation is not equivalent to typing error, which includes scoring errors, allelic dropout, stutter, and null alleles, often reaching 1%–15% across loci (Pompanon et al. 2005). To model these processes separately, we first generated the entire population with mutation incorporated. In both mutation and genotyping error models, when an allele substitution occurred, the replacement allele was sampled in proportion to its population frequency, thereby reflecting allelic preference rather than uniform random substitution. Genotyping errors were then applied post hoc to the STR data, since these errors arise during laboratory scoring and are not inherited across generations. The mating system was based on the random union of gametes from male and female gametic pools. Each female was considered to have one or two offspring with probabilities of 0.95 and 0.05, respectively. This design also allowed the occurrence of full‐sibling offspring, a common situation in many animal populations.

The simulation was conducted in two main steps. First, historical generations were simulated to establish desirable allele frequencies. Second, recent population structures were generated. In the initial generation (*g* = 0) of the historical population, we simulated 2000 individuals with uniform allele frequencies across all STRs. A total of 50 historical generations were simulated, with the number of individuals gradually decreasing from 2000 to 200 from generation 0 to 50. We simulated 50 historical generations as a balance between biological realism and computational efficiency: This duration was sufficient to stabilize allele frequency distributions and minimize founder effects, while additional generations did not materially change the results. Following the historical population simulation, 10 recent populations were simulated, encompassing generations 0 to 10. Generation 0 of the current population consisted of selected individuals from generation 50 of the historical population. In our simulations, the allele set was fixed at the start, and no novel alleles outside this set were introduced in subsequent generations. After simulating the current population, typing errors, either constant or variable for each locus based on the study design, were applied to the STR data. For simulations assuming constant genotyping error, we used an error rate of 1% across all loci. For simulations with variable error, the per‐locus error rate was drawn between 1% and 6% to reflect heterogeneity among loci. The number of individuals per generation in the current population was kept constant at 200. Ultimately, we had 2200 individuals (including 200 from generation 0) with both pedigree and STR information. The last two generations (*g* = 9 and 10) were used for paternity testing and assignment. The simulation process was repeated 10 times to mitigate random sampling error and ensure the reliability of the statistical estimates.

### 2.6 Calculation of Typing Errors for Each Locus

After simulating the population, typing errors for each locus were calculated to replicate real‐world conditions where the exact values of typing errors are unknown. A typing error is defined as the substitution of the true paternal or maternal allele at a specific locus in an individual with a random allele. These errors can occur in the genotypes of the alleged father, mother, or offspring. To calculate typing errors, we identified mismatches between offspring and both parents across the entire population, excluding the last two generations, which were reserved for accuracy assessment. Initially, we selected mother–offspring and father–offspring pairs with only one typing error. Subsequently, the total number of errors for each locus was determined by summing the identified paternal and maternal errors. We utilized an in‐house script written in R version 4.4.1 to calculate the typing errors.

### 2.7 Find the Significance Criteria

We utilized CERVUS software version 3.0.7 to determine the significance criteria of Δ, using allele frequencies derived from the current population (Kalinowski et al. 2007; Marshall et al. 1998). A total of 10,000 tests were conducted to generate distributions of Δ for each simulated population individually. The Δ values were calculated at confidence levels of 95% and 99%, representing the thresholds for significant assignment accuracy. Various population structures, including the relatedness among true parents, the population‐wide inbreeding rate, and the proportion and magnitude of the relatedness of candidate fathers to the true father, were calculated for each simulated population and incorporated into the CERVUS paternity assignment. However, when applying the CERVUS algorithm for paternity testing, the Δ values were calculated under the assumption that no population structure or candidate male representation was accounted for, as this analysis assumes no male candidate is represented.

For the Pairwise method, significance criteria for the LOD score were established based on its simulated distribution for the true father. Critical LOD values corresponding to *p* values of 0.05 and 0.01 were determined, ensuring that 95% and 99% of the simulated LOD scores exceeded these threshold values, respectively.

### 2.8 Accuracy Calculation

To evaluate the accuracy of two methods, we calculated the accuracy metric using true‐positive (TP) and true‐negative (TN) rates. Although false‐positive and false‐negative rates can provide complementary information (Christie et al. 2013), in our comparison of Pairwise and CERVUS we focused on true‐positive and true‐negative rates, which directly illustrate the tradeoff between maximizing correct assignments and minimizing erroneous ones. This evaluation was conducted on the last two generations from the current simulated population (*n* = 400). For the paternity test, the odds (LOD) score for the true father was calculated. As our simulation included 50 candidate males per generation, an equal number of random candidate males with LOD scores were simulated. In our method, we compared the calculated LOD scores for both the true father and the candidate males against a predefined significance threshold. If the LOD score for the true father exceeded this threshold and all other candidate males had lower LOD scores, the true father was identified as the biological father. However, if the true father’s LOD score was below the threshold, or even if it exceeded the threshold but another candidate male had a higher LOD score, paternity was left unassigned. The same procedure was applied using the CERVUS methodology, but with Δ values and their corresponding significance criteria. The proportion of correctly identified fathers was represented as the TP calculated for the paternity test. The TN was calculated similarly to the TP, but with the true father removed from the candidate pool. The proportion of tested individuals where random (for the paternity test) or candidate males (for assignment) did not exceed their corresponding significance criteria was considered TN. Higher values of both TP and TN indicate greater accuracy of the model.

## 3 Results

All simulations and analyses for the Pairwise paternity assignment method were conducted using custom R scripts, which are publicly available at: https://github.com/mahmood225/PairwisePaternity.

### 3.1 Pairwise vs. CERVUS Methods

**FIGURE 3:**
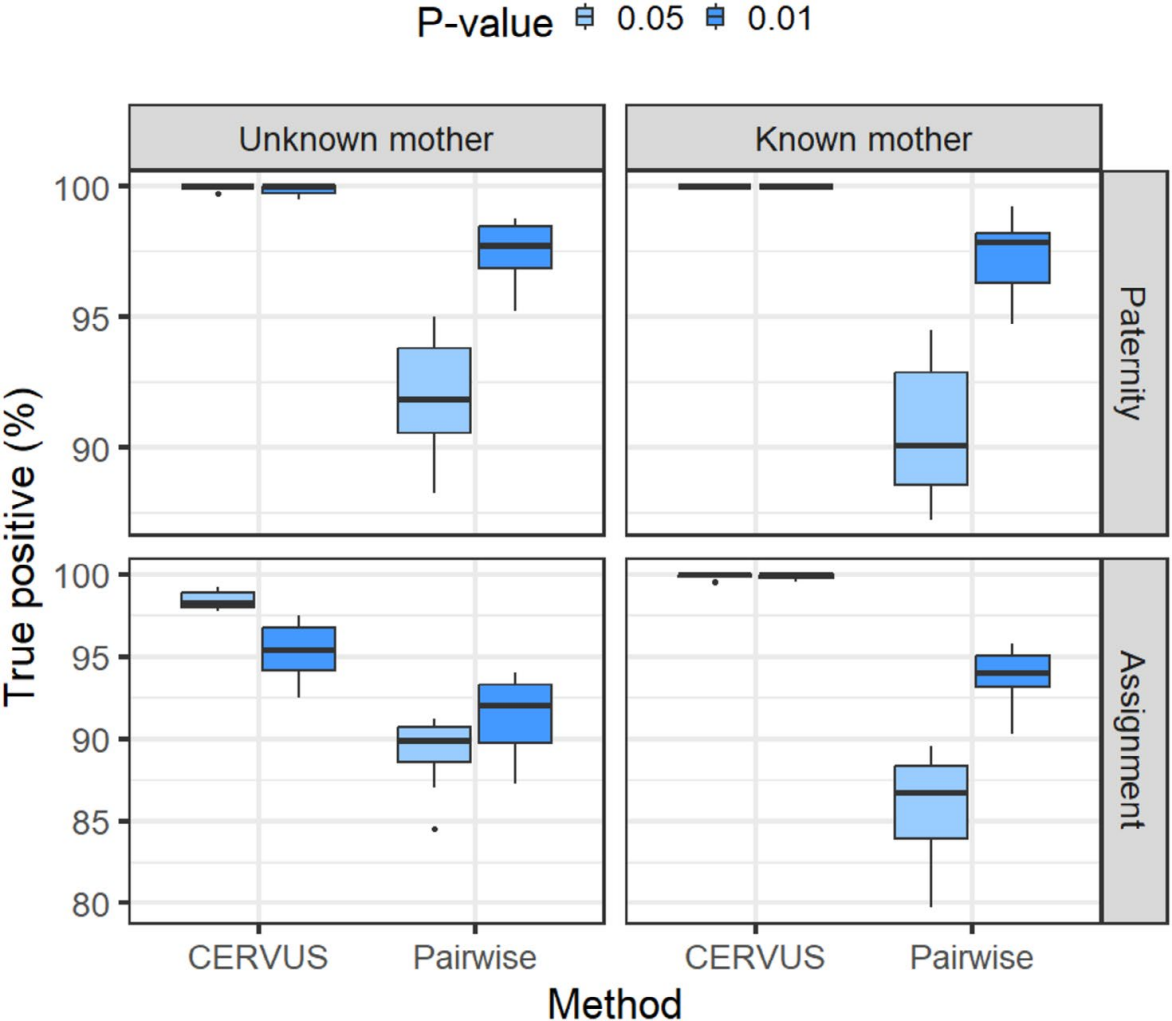
Plot showing the true‐positive (TP) rates achieved by the Pairwise and CERVUS methods in both paternity testing and assignment scenarios. The analysis considers conditions where the maternal genotype is either known or unknown.

The accuracies of paternity testing and assignment were evaluated using TP and TN calculations for both the CERVUS and Pairwise algorithms, based on a dataset of 400 simulated offspring with known parents. Overall, the CERVUS algorithm demonstrated higher TP accuracy compared with the Pairwise method (Figure 3). For the Pairwise algorithm, a *p* value of 0.01 yielded the highest TP accuracy for paternity testing, with an average of 97.60% (±1.12%) when the STR genotype of the mother was unknown, and 97.33% (±1.34%) when it was known. In contrast, for the CERVUS algorithm, a cutoff point at *p* value < 0.05 resulted in the highest TP accuracy for paternity testing, with an average of 99.85% (±0.21%) when the mother was unknown, and 100% (±0%) when the mother was known. Both methods showed a significant reduction in accuracy from paternity testing to assignment (*p* value < 0.01). Specifically, when the mother was unknown, the TP accuracy decreased by 2.68% (6.13%) and 1.53% (4.58%) for the Pairwise and CERVUS algorithms with *p* values of 0.05 (0.01), respectively. When the mother was known, the TP accuracy for the Pairwise algorithm decreased by 4.65% (3.53%) with *p* values of 0.05 (0.01), while the CERVUS algorithm showed minimal change, with reductions of approximately 0.1% and 0.13% for *p* values of 0.05 or 0.01, respectively, maintaining nearly 100% accuracy.

**FIGURE 4:**
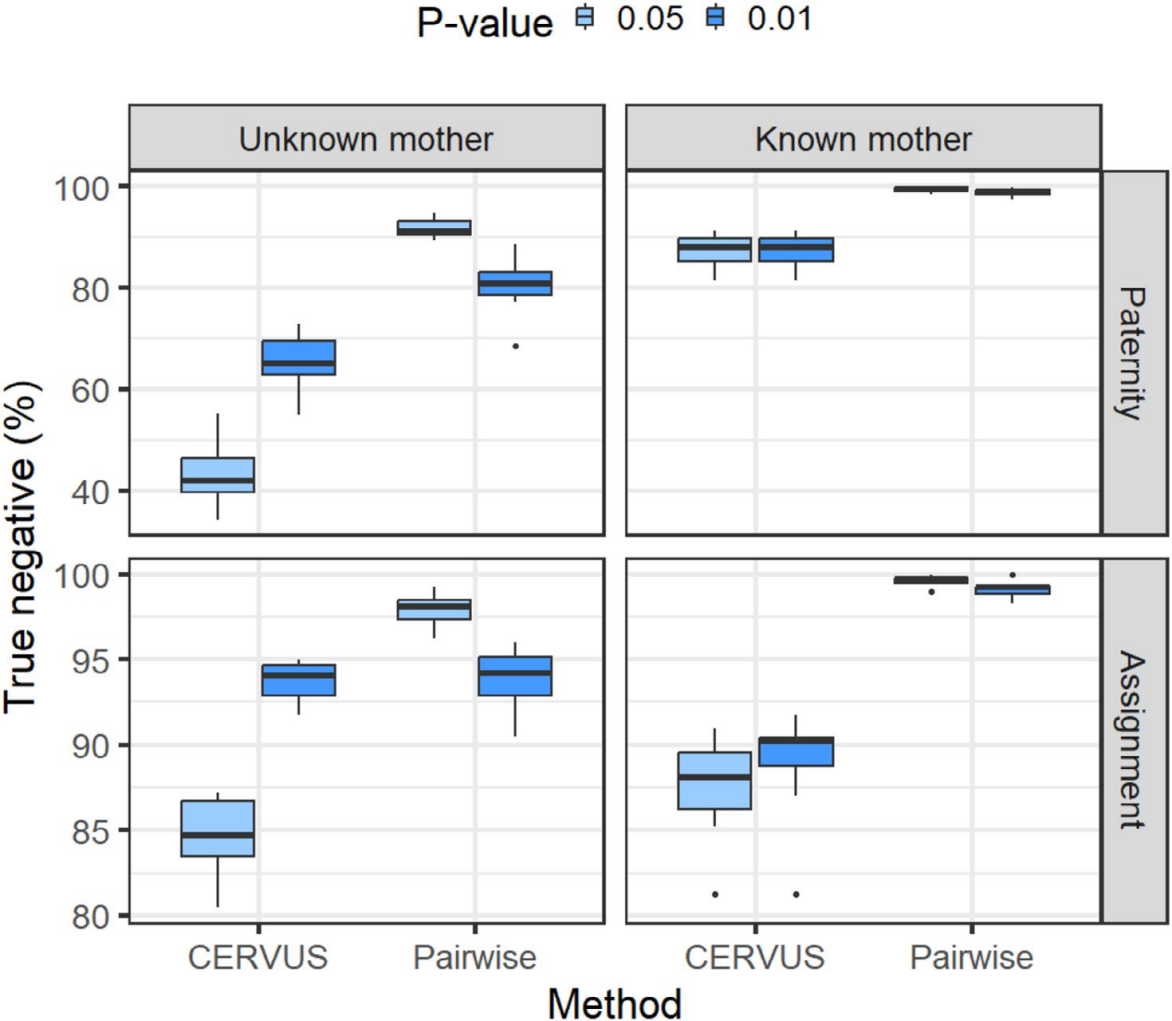
Plot illustrating the accuracy of true‐negative results achieved by the Pairwise and CERVUS methods in both paternity testing and assignment scenarios, with conditions where the maternal genotype is either known or unknown.

The accuracy calculated based on TN rates indicated that the Pairwise method demonstrated significantly higher accuracy compared with the CERVUS method (Figure 4). In 10 repeated simulations with known maternal genotype, the Pairwise method correctly identified 99.43% ± 0.53% and 98.85% ± 0.84% of random (for paternity test) as nonparents, with *p* values of 0.05 and 0.01, respectively. For candidate males (for assignment), it achieved accuracies of 98.85% ± 0.84% and 99.1% ± 0.46% at the same *p* value thresholds. In the CERVUS method, these accuracy estimates were significantly reduced to 87.28% ± 3.24% (for both *p* values) and 87.58% ± 2.88% and (89% ± 3.03%) for paternity and assignment, respectively. When the maternal genotype was unknown, the Pairwise method for assignment yielded a TN rates of 93.78% ± 1.9% for a *p* value of 0.01 and 97.95% ± 0.96% for a *p* value of 0.05. However, the Pairwise method for paternity testing produced relatively lower TN rates of 80.43% ± 5.24% and 91.7% ± 1.97%. The estimated TN rates for the CERVUS method were significantly lower in paternity testing compared with the Pairwise method, with values of 65.4% ± 5.65% and 43.45% ± 6.32%. In assignment, the CERVUS method exhibited slightly lower performance compared with the Pairwise method, with TN rates of 93.78% ± 1.23% for a *p* value of 0.01 and 84.68% ± 2.25% for a *p* value of 0.05.

### 3.2 Effect of Relationship Between the True Father and Candidate Male

**FIGURE 5:**
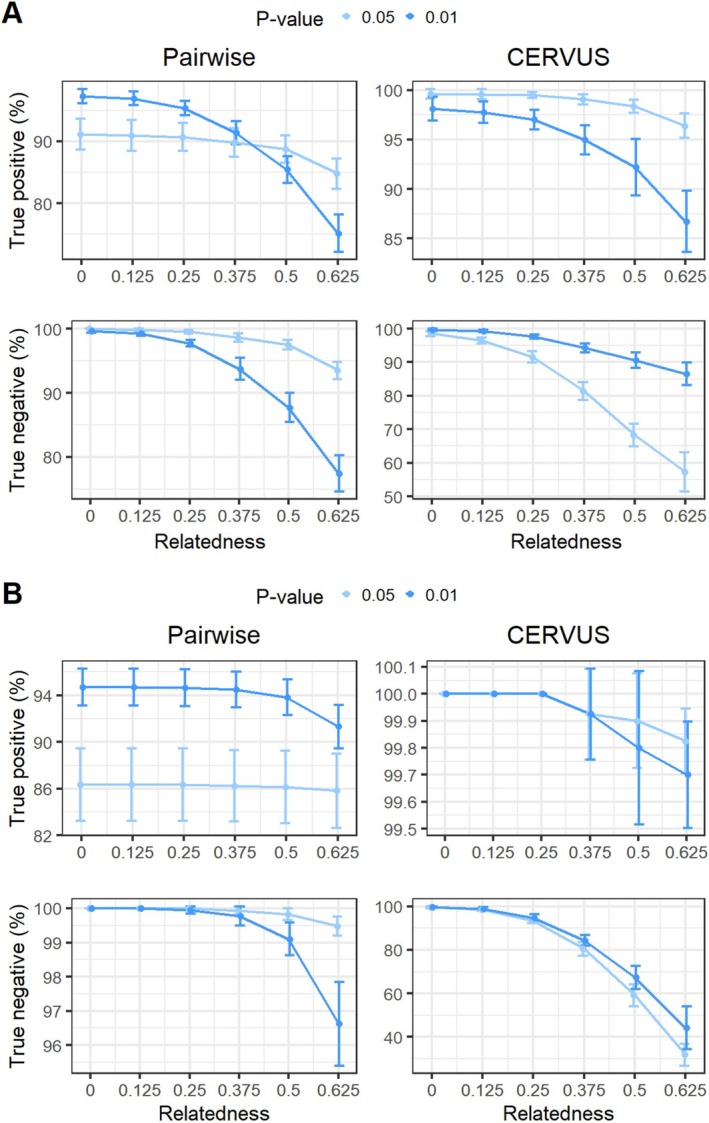
Impact of introducing related candidate males on the true‐positive and true‐negative rates of parentage assignments is illustrated for scenarios where the maternal genotype is unknown (A) and known (B).

The relationship between the pool of male candidates and the mother, father, or offspring can influence the accuracy of the assignment test (Marshall et al. 1998). To assess this effect, we simulated 50 candidate males (the same number used as fathers in each generation) with different proportions of genotype similarity to the true father. This simulation attempts to imitate the relatedness degree ranging from 0 (unrelated) to 0.625 (highly related) (Figure 5). To achieve this, proportions of 0, 0.125, 0.25, 0.375, 0.5, and 0.625 of the simulated STRs for each candidate were randomly selected and unselected using the “sample()” function in R, with the probability set to degree of relatedness and 1—relatedness, respectively, to apply the similarity between the candidate and the true father. For each randomly selected STR, one of the two alleles of the true father was selected and assigned to the candidate, while the other allele was randomly selected based on the allele frequency of the population. Both TP and TN metrics in paternity assignments decreased as relatedness increased, regardless of whether the mother's genotype was known or unknown. With unknown mothers, both methods showed a similar pattern of decreasing TP rates. However, the decrease in TN rates was more obvious for CERVUS compared to Pairwise. This suggests that the absence of maternal information has a greater impact on CERVUS, leading to a higher likelihood of incorrect assignment of a father when the candidates are more closely related to the true father and the true father is not included among the candidates. Knowledge of the maternal genotypes mitigated the impact of relatedness between the true father and candidate males on TP rates. However, when TN rates were used as the accuracy criterion, maternal genotypes did not prevent a decrease in TN using the CERVUS method, although they were beneficial when the Pairwise method was employed.

### 3.3 Typing Error Assumption

**FIGURE 6:**
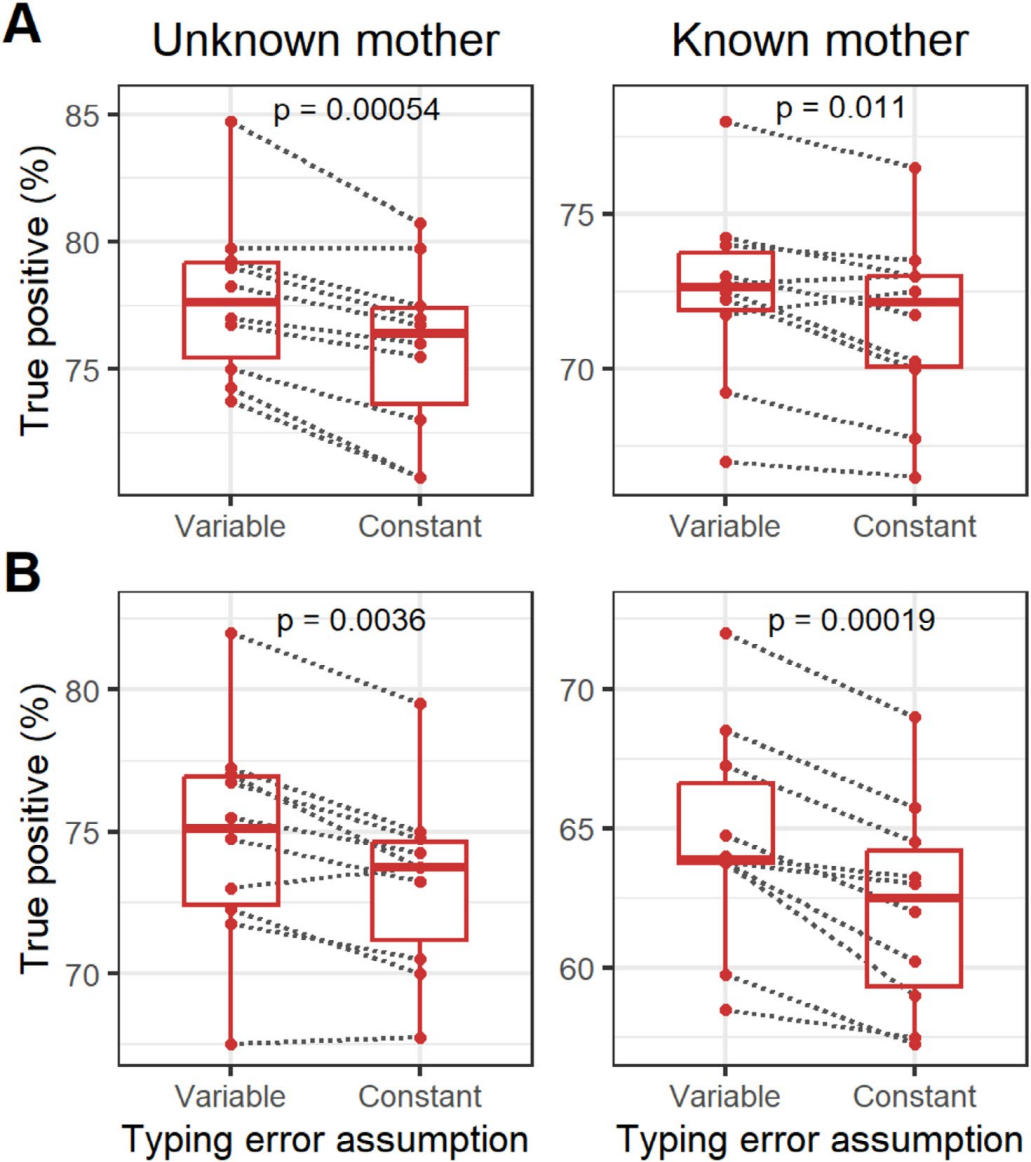
Boxplot illustrates the true‐positive accuracy achieved using the Pairwise method, under the assumption of either variable or constant typing errors across different loci for paternity testing (A) and assignment (B). The means of two measurements obtained from the same simulation were compared using a paired *t*‐test, with the corresponding *p* values (*p*) displayed at the top of each plot.

Overall, considering variable typing errors across different loci led to an increase in true‐positive (TP) rate accuracy compared to constant typing errors (Figure 6). This enhancement was particularly evident in both paternity tests and paternity assignments. For paternity tests, when the maternal genotype was unknown and constant typing errors were assumed, the TP rate was 75.78% ± 3.4%. By adding variable typing errors, the accuracy increased to 77.78% ± 3.24%, reflecting a significant improvement of 2.00%. With assigning the true fathers, the improvement was approximately 1.5%, increasing from 73.25% ± 3.24% with constant errors to 74.78% ± 3.93% with variable errors. The highest improvement was observed in paternity assignments when the maternal genotype was known, with an increase of 2.45%; the values increased from 62.15% ± 3.75% to 64.6% ± 3.95%. When the maternal genotype was known, the TP improved by 1%, from 71.47% ± 2.93% to 72.47% ± 2.93%, under variable typing errors across different loci.

**FIGURE 7:**
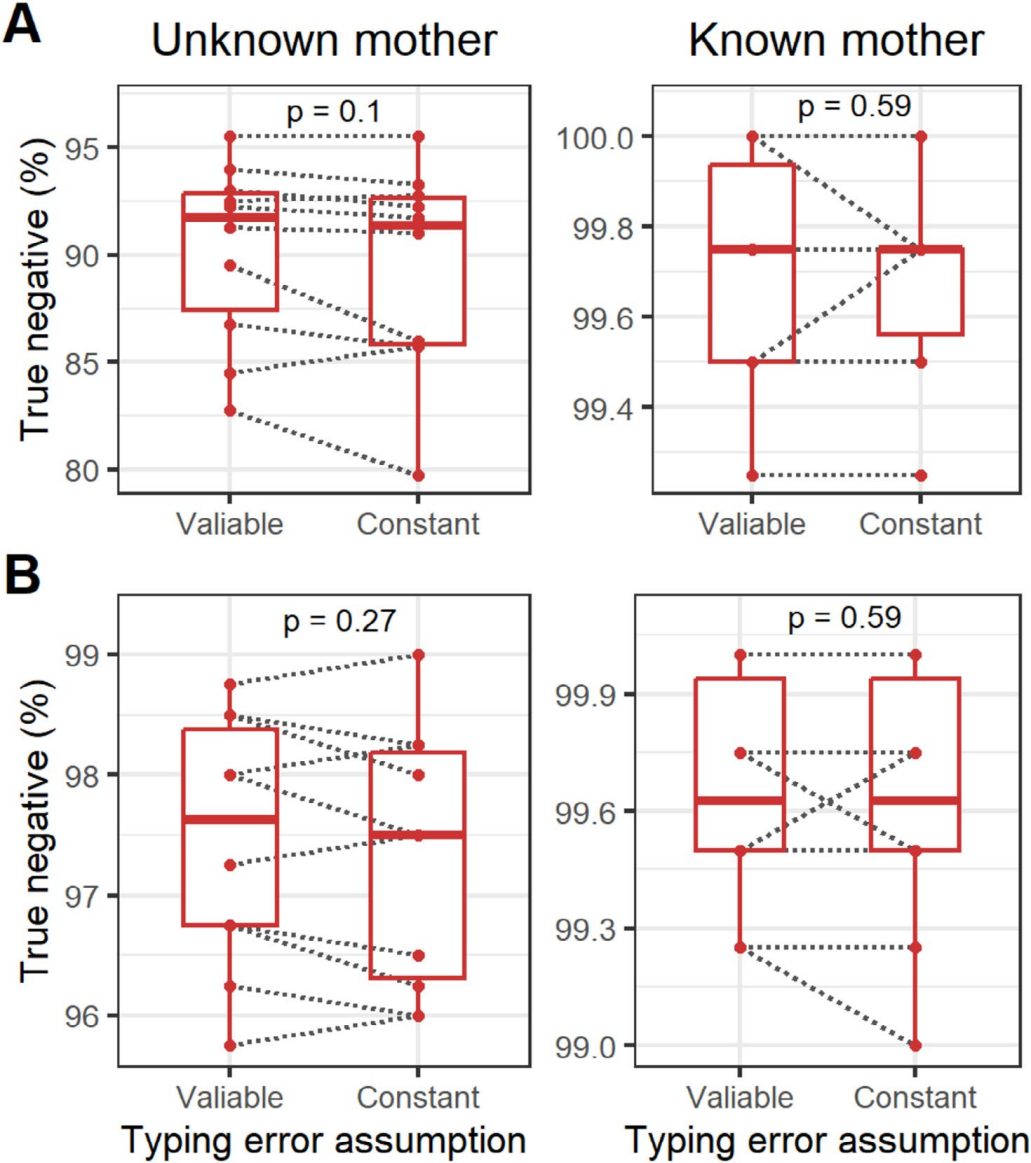
Boxplot illustrates the true‐negative accuracy achieved using the Pairwise method, under the assumption of either variable or constant typing errors across different loci for paternity testing (A) and assignment (B). The means of two measurements obtained from the same simulation were compared using a paired *t*‐test, with the corresponding *p* values (*p*) displayed at the top of each plot.

Interestingly, the improvement in TP did not significantly affect the true‐negative (TN) rate (Figure 7). In other words, accounting for variable typing errors across different loci improved the performance of paternity analysis by increasing the probability of correctly identifying the true parent without increasing the likelihood of incorrectly assigning a parent.

## 4 Discussion

In this study, we introduced a novel method called the “Pairwise” approach for calculating the significance criteria for the likelihood of paternity, which enhances the performance of paternity tests and assignments by increasing the true‐negative rate compared to the critical values of Δ (Kalinowski et al. 2007; Marshall et al. 1998). Our approach provides a critical value for each parent (or pair of parents, if both paternal and maternal genotypes are known) individually, making the value specific to each alleged parent. This case‐specific feature is particularly useful in ecological and conservation contexts where the true parent may be absent from the candidate pool, the maternal genotype is unknown, or relatedness among candidate males is high. Our suggested method works based on the concept that each paternity test has its own hypothetical distribution due to the alleles present in the parental and offspring genotypes. This case‐by‐case hypothetical distribution can also be influenced by the presence of different missing genotypes in various individuals, which can be accounted for using the Pairwise method. Our simulation system for calculating the critical value offers a significant advantage over previously introduced methods (Kalinowski et al. 2007; Marshall et al. 1998). The Pairwise method can be performed without any prior information, such as the number of candidates and their relationship with the offspring or true parents, about the candidate being tested to determine whether the alleged father or mother is the biological parent. However, for paternity assignment, the presentation of candidate males or females and their genotype information remains essential. In a comparative analysis, we showed that our novel method significantly improves the accuracy and reliability of paternity testing by providing individualized critical values and accommodating various genotypic scenarios, thereby advancing the field of genetic testing and its applications. In these common field situations, the ability of Pairwise to improve true‐negative accuracy and reduce false assignments provides clear practical value.

Paternity inference relies on the relatedness structure between offspring and parents. However, developing a method for paternity assignment that remains unaffected by the population's relatedness structure is both challenging and highly desirable. Marshall et al. (1998) introduced a simulation methodology for calculating the critical value using Δ for paternity inference, which demonstrated robustness even in the presence of close relatives among the parents and candidates. However, another study showed that that CERVUS performance declines when closely related individuals are present among candidate parents and when the number of sampled parents is unknown, as these conditions increase false assignments (Christie et al. 2013). In concordance with these findings, our simulation—examining the relationship between the true father and candidate males—demonstrated that the CERVUS algorithm is more sensitive to relatedness when one parent (in our case, the maternal genotype) is known. In contrast, the Pairwise method exhibited reduced sensitivity to such similarities, consistently achieving higher true‐negative accuracy, particularly when the maternal genotype was unknown or the true father was absent. Although CERVUS generally produced higher true‐positive rates, this difference highlights a tradeoff: CERVUS maximizes correct assignments when the true parent is present, whereas Pairwise minimizes false assignments when the true parent is missing. Thus, the choice of method depends on study context—CERVUS is advantageous when the true parent is likely sampled, while Pairwise is more reliable under incomplete sampling or missing parental information. The superiority of our method was particularly evident when the true parent was not among the examined candidates, as it introduced a smaller number of nonparent males as the true parent.

In conclusion, our innovative paternity assignment method not only addresses the inherent challenges posed by relatedness structures within populations but also significantly enhances the accuracy of paternity inference. By minimizing the introduction of nonparent males as true parents, especially in scenarios where the actual parent is absent among the candidates, our approach sets a new benchmark for reliability and precision in genetic studies. In practical applications, known half‐ and full siblings of the offspring can be excluded from the candidate pool prior to analysis, which ensures that the candidate set reflects only putative parents and avoids confounding effects from sibling genotypes. A potential concern is whether the higher true‐negative performance of Pairwise simply reflects outcomes comparable to those obtained by traditional exclusion methods. However, exclusion‐based approaches only rule out candidates when allelic mismatches occur, and they do not provide statistical thresholds for significance. In contrast, Pairwise generates LOD‐based, trio‐specific significance criteria that remain robust in the presence of genotyping error or missing data. This means Pairwise can correctly reject false candidates even when mismatches are obscured by error, while still providing a quantifiable level of statistical confidence in the assignment decision. Finally, the proportion of the population sampled is also a critical factor influencing assignment accuracy: Incomplete sampling reduces true positives and makes improvements in true‐negative control particularly valuable.

In addition to CERVUS, other programs such as COLONY have been developed to address relatedness and pedigree complexity by jointly inferring sibship and parentage while incorporating locus‐specific error rates (Jones and Wang 2010). COLONY is particularly effective when comprehensive sampling enables reconstruction of full family structures. However, its reliance on pedigree‐wide inference and higher computational demands can limit its utility in ecological studies where researchers focus on a restricted set of candidate parents or where maternal genotypes are missing. By contrast, the Pairwise approach is designed to be lightweight and targeted: It generates trio‐specific significance thresholds through forward–backward simulations, directly improving the control of false assignments without requiring pedigree‐wide inference. Thus, Pairwise complements but does not replace COLONY, offering ecologists a flexible option when focused, case‐by‐case paternity resolution is required.

Bayesian approaches offer a robust alternative to traditional paternity inference methods, providing flexible trio‐based models that estimate genetic relationships while accounting for uncertainty and prior information (Christie et al. 2013; Goldgar and Thompson 1988). By using genetic marker data, Bayesian methods enable estimation of the posterior probability of genetic relationships—such as paternity—without relying on strict null hypotheses, and Monte Carlo simulations have shown their effectiveness in distinguishing true fathers from closely related or unrelated individuals with high precision (Goldgar and Thompson 1988). These approaches have proven effective in handling genotyping uncertainty, offering robust parentage inference by minimizing false assignments regardless of the genotyping error rate. SOLOMON, a Bayesian method, has been shown to outperform CERVUS by maximizing correct assignments while significantly reducing false positives, particularly when marker data is limited (Christie et al. 2013). In our study, we addressed this same issue by adjusting the CERVUS algorithm, which led to a notable reduction in false assignments through trio‐specific simulations and variable error modeling.

Mutations and laboratory errors can lead to discrepancies in genotyping, resulting in deviations from Mendelian inheritance patterns between parental and offspring genotypes. Accounting for these mismatches under analytical conditions in parentage analysis has proven to be highly beneficial for accurately assigning or excluding parentage with a certain degree of confidence (Morrissey and Wilson 2005). However, the presence of typing errors generally has a negligible impact on the accuracy of assignments. Nonetheless, an increase in genotyping errors can lead to a 1%–3% decrease in accuracy (Harrison et al. 2013). Mendelian inheritance with a 1% genotyping error is typical for STR loci (Pompanon et al. 2005). If such error rates are prevalent on a large scale, they are likely to be a significant cause of mismatches between offspring and their true parents. Various methods for parentage testing or assignment have been proposed, incorporating different analytical conditions to address typing errors. However, to date, all these methods assume a constant typing error value across different loci (Christie 2010; Gerber et al. 2003; Kalinowski et al. 2007; Marshall et al. 1998). In reality, it is more plausible that genotyping errors vary from one locus to another, particularly for STR data (Bonin et al. 2004; Creel et al. 2003; Slate et al. 2000). By adjusting the methodology introduced by Kalinowski et al. (2007) for calculating the LOD, we were able to account for variable typing errors across loci. Our approach is also conceptually related to Famoz (Gerber et al. 2003), which provided more flexibility than CERVUS but is no longer supported; however, the Pairwise framework extends this line of development by incorporating locus‐specific genotyping error, trio‐specific thresholds, and explicit treatment of relatedness, and is implemented in R for long‐term usability. Using this approach, we demonstrated for the first time that assuming variable typing errors between different loci can significantly improve the accuracy of true father assignment without increasing false assignments. Our method underscores the importance of accounting for variable genotyping errors across loci in parentage analysis. This approach not only improves the control of false assignments by enhancing true‐negative accuracy but also provides a more robust framework for genetic testing and research. In general, the primary advantage of employing adjusted likelihood equations to account for variable typing errors is the reduction in erroneous parentage assignments and greater reliability of results, even though the absolute number of true parent assignments may not increase relative to CERVUS.

While our work indicates the consistent advantages of incorporating variable genotyping errors in paternity tests, it is crucial to note that the genotyping error model used here is somewhat primitive when compared to real‐world settings. For example, our model assumed a random genotype replacement for any loci exhibiting errors, which is an unlikely scenario since certain types of errors are more prevalent than others (Bonin et al. 2004). Nonetheless, it provides a tractable framework to evaluate the effect of variable locus‐specific error rates on assignment accuracy. Advanced likelihood equations have been formulated to address more realistic scenarios of genotyping errors (Gill et al. 2000; Kalinowski et al. 2006; Sobel et al. 2002; Wang 2018). Several studies have compared CERVUS and COLONY in different ecological systems (Harrison et al. 2013; Karaket and Poompuang 2012; Thow et al. 2022; Walling et al. 2010), but these evaluations have focused primarily on overall assignment performance and pedigree reconstruction. Future extensions of the method could incorporate more biologically realistic error models to further assess robustness under empirical conditions. By contrast, sophisticated likelihood‐based error models have not yet been systematically compared with the simpler paternity analysis framework of Kalinowski et al. (2007) to determine whether they yield more accurate paternity estimates under variable error conditions. While this method is straightforward, its effectiveness can be enhanced by incorporating preference allelic substitution into the likelihood equation. Thus, further investigation into these areas is warranted. Finally, while we focused on parameter combinations that reflect common field conditions (10 STR loci, 1%–6% locus‐specific genotyping error, incomplete sampling, and varying relatedness), a broader exploration of the parameter space (e.g., number of loci, allele frequency spectra, sampling fraction, and panel heterogeneity) represents an important direction for future work.

STRs have been widely used for parentage analysis, especially in species with high genetic variability, such as many fishes (DeWoody and Avise 2000). However, their application can be limited by low polymorphism in some species, labor‐intensive marker development, and subjective allele scoring (Flanagan and Jones 2019). With advances in genomic technologies, SNPs have emerged as a practical alternative, offering easier automation, lower mutation rates, and reliable performance—even in low‐diversity systems. Although our study was based on STR data, the Pairwise framework can be directly applied to SNP panels, where the large number of independent loci and generally lower genotyping error rates are expected to increase assignment power, potentially enhancing the benefits of trio‐specific thresholds. Notably, recent tools such as the apparent R package have demonstrated high accuracy in identifying parent–progeny relationships using genome‐wide SNP data, even in the complete absence of prior pedigree information (Melo and Hale 2019). Apparent uses the genomic relationship likelihood (GRL) method, which was shown to be both fast and highly accurate (~99%) for parentage assignment using dense SNP data, without requiring knowledge of genotyping error or call rates across loci (Grashei et al. 2018). In comparative tests, apparent achieved 100% accuracy when minimal generational information was provided, matching the performance of CERVUS under the same conditions, while outperforming it in fully unguided scenarios. Our approach, while developed using STR data, is flexible and can be adapted for SNP‐based parentage analysis. Future studies should explore its application across different marker types and diverse population structures to further validate its utility.

## 5 Conclusion

In conclusion, our study introduces a novel method for calculating the likelihood of paternity, enhancing the performance of the paternity‐associated tests. Our suggested approach, by accounting for parents' unique hypothetical distribution, offers a significant advantage over previously introduced methods. By addressing the inherent challenges posed by relatedness structures within populations, our method significantly improves the accuracy of paternity inference. Furthermore, our findings underscore the importance of accounting for variable genotyping errors across loci in parentage analysis. This approach does not increase the number of true parent assignments compared to CERVUS; rather, it improves reliability by reducing false assignments and enhancing true‐negative accuracy, thereby providing a more robust framework for genetic testing and research. Overall, our innovative paternity assignment method sets a new benchmark for reliability and precision in genetic studies. By minimizing the introduction of nonparent males as true parents, especially in scenarios where the actual parent is absent among the candidates, our approach significantly advances the field of genetic testing and its applications.

## Author Contributions

**Mahmoud Amiri Roudbar:** conceptualization (lead), data curation (lead), formal analysis (lead), methodology (lead), project administration (lead), software (lead), supervision (lead), visualization (lead), writing – original draft (lead). **Seyedeh Fatemeh Mousavi:** conceptualization (supporting), data curation (supporting), formal analysis (supporting), methodology (supporting), visualization (supporting), writing – original draft (supporting). **Mahdi Akbarzadeh:** methodology (supporting), validation (supporting), writing – review and editing (supporting). **Sabrina H. Brounts:** validation (supporting), writing – review and editing (supporting). **Mehdi Momen:** conceptualization (supporting), formal analysis (supporting), methodology (supporting), validation (lead), writing – review and editing (lead).

## Conflicts of Interest

The authors declare no conflicts of interest.

## Data Availability

The R codes utilized in this study are publicly available on GitHub at the following repository: https://github.com/mahmood225/PairwisePaternity.
